# Under-detection of blood culture-positive enteric fever cases: The impact of missing data and methods for adjusting incidence estimates

**DOI:** 10.1371/journal.pntd.0007805

**Published:** 2020-01-16

**Authors:** Merryn Voysey, Dikshya Pant, Mila Shakya, Xinxue Liu, Rachel Colin-Jones, Katherine Theiss-Nyland, Nicola Smith, Shrijana Shrestha, Buddha Basnyat, Andrew J. Pollard, Virginia E. Pitzer

**Affiliations:** 1 Oxford Vaccine Group, University of Oxford, Oxford, United Kingdom; 2 NIHR Oxford Biomedical Research Centre, Oxford, United Kingdom; 3 Oxford University Clinical Research Unit, Patan Hospital, Kathmandu, Nepal; 4 Department of Epidemiology of Microbial Diseases, Yale School of Public Health, New Haven, United States of America; Johns Hopkins Bloomberg School of Public Health, UNITED STATES

## Abstract

**Background:**

In surveillance for typhoid fever, under-detection of cases occurs when patients with fever do not seek medical care, or seek medical care but do not receive a blood test. Missing data may result in incorrect estimates of disease incidence.

**Methods:**

We used data from an ongoing randomised clinical trial of typhoid conjugate vaccine among children in Nepal to determine if eligible patients attending our fever clinics who did not have blood taken for culture had a lower risk of disease than those who had blood drawn. We assessed clinical and demographic predictors of having blood taken for culture, and predictors of culture-positive results. Missing blood culture data were imputed using multiple imputations.

**Results:**

During the first year of surveillance, 2392 fever presentations were recorded and 1615 (68%) of these had blood cultures. Children were more likely to have blood taken for culture if they were older, had fever for longer, a current temperature ≥38 degrees, or if typhoid or a urinary tract infection were suspected. Based on imputation models, those with blood cultures were 1.87 times more likely to have blood culture-positive fever than those with missing data.

**Conclusion:**

Clinical opinion on the cause of the fever may play a large part in the decision to offer blood culture, regardless of study protocol. Crude typhoid incidence estimates should be adjusted for the proportion of cases that go undetected due to missing blood cultures while adjusting for the lower likelihood of culture-positivity in the group with missing data.

## Author summary

Typhoid fever is a serious illness caused by a bacterial infection in the blood. Typhoid spreads through contaminated water sources, poor sanitation or poor hygiene, and occurs most often in low-resource environments, with children being most at risk. It is hard to determine how common typhoid infections are in low-resource setting as there are many pathogens that can cause diseases with similar characteristics, and not all patients present to health-care facilities. The culture test to detect the presence of bacteria in the blood is poorly sensitive, costly to perform, and not available in all facilities. Therefore, true disease cases often go undetected, resulting in an underestimation of the true burden of disease. We examined factors associated with blood culture collection in fever patients attending typhoid surveillance clinics in Kathmandu, Nepal. We aimed to determine whether those who did not have a blood culture were different in terms of clinical and demographic characteristics to those who did have blood taken for culture and, additionally, whether they were more or less likely to test blood culture positive for typhoid fever. We provide estimates of inflation factors that can be used to adjust the crude incidence rate to account for the under-detection of typhoid cases.

## Introduction

Typhoid and paratyphoid fever are enteric infections caused by the bacteria *Salmonella enterica* serovars Typhi (*S*. Typhi) and Paratyphi (*S*. Paratyphi). The World Health Organisation recommends the use of typhoid conjugate vaccine (TCV) for the control of typhoid fever, particularly in countries with high incidence or a high burden of antimicrobial resistant *S*. Typhi.[1] Blood culture surveillance programmes are essential for understanding which countries have a high burden of disease. Surveillance programmes aim to estimate the incidence of disease by detecting all cases of blood culture-positive typhoid fever in a given population or at sentinel surveillance sites, but the quality of surveillance can vary substantially between countries.[2] If under-detection of cases occurs, then disease incidence will be under-estimated. Missed cases can occur when patients with fever do not seek medical care, attend a clinic that is not part of the study surveillance programme, or seek medical care but do not receive a blood culture test required for diagnosis. In addition, blood culture tests have poor sensitivity[3], especially in settings where self-treatment with widely available antibiotics may occur prior to blood culture.

Crude typhoid incidence estimates can be adjusted for various factors that affect the under-detection of cases.[4] As a minimum, crude incidence estimates are often adjusted for the poor sensitivity of blood culture tests;[5] among those with typhoid fever, only around 60% of individuals who receive a blood culture are expected to test positive.[3] Additionally, incidence can be adjusted to account for missed cases using inflation factors based on data from community surveys or other sources of information on health-care seeking behaviour.[6, 7] However, incidence estimates also need to be adjusted for the number of patients who have typhoid fever meeting pre-specified eligibility criteria and attend a surveillance facility but do not receive a blood culture. The reasons for these missed cases are often unknown and may vary between sites.

Analysts sometimes assume that the person who meets pre-specified eligibility criteria but does not have a blood culture performed is just as likely to be a true disease case as the person who attended the same facility and did have a blood culture taken.[8] In statistical terms, this type of missing data are referred to as data that are “missing-completely-at-random” (MCAR).[9] When modelling risk factors for disease, MCAR data can be safely ignored without compromising the validity of statistical models. This assumption may be suitable when the reason a blood is not taken is due to limited laboratory capacity or staffing restrictions rather than due to factors associated with the patient themselves.

An alternative understanding of fever patients who attend a facility, meet the pre-specified screening criteria, but do not receive blood culture, is that such individuals may be more or less likely to have typhoid fever. The probability of blood being drawn for culture may be related to clinical or other factors that can be measured at the time of their presentation to the fever clinic. This type of missing data, where missingness is related to other measurable factors such as the patient’s age or duration of fever, are referred to as data which are “missing-at-random” (MAR). Analysing MAR data without taking into account the effect of the missing data can introduce bias.[10]

When estimating typhoid incidence, it is important to understand whether missing blood culture data are MAR or MCAR. Disease incidence estimates may be over- or under-estimated if assumptions about missing data do not hold true.

We examined the factors associated with blood culture collection in fever patients attending typhoid surveillance clinics in Kathmandu, Nepal. We aimed to determine whether those without blood cultures performed were different in terms of clinical and demographic characteristics to those with blood cultures performed and, additionally, whether they were more or less likely to test blood culture positive for typhoid fever. We provide estimated inflation factors that can be used in the calculation of adjusted incidence.

## Methods

TyVAC-Nepal is a double-blind randomised controlled trial of the efficacy of typhoid conjugate vaccine for the control of typhoid fever.[11, 12] We randomized 20,019 children aged 9 months to <16 years in the Lalitpur Metropolitan Area of Kathmandu, Nepal, to receive either TCV or Group A meningococcal conjugate vaccine (MenA) between November 2017 and April 2018. The primary outcome is the incidence of blood culture-confirmed typhoid fever during two years of follow-up, assessed using passive as well as active surveillance. For the passive surveillance, trial participants who experience fever are encouraged to attend one of the 18 community clinics or one tertiary-care hospital, which also provides outpatient and secondary care services to the local community. Those who seek care are eligible for blood culture if they have a self-reported fever of at least 2 days in duration or a current temperature of at least 38 degrees Celsius. Clinical staff were trained to take blood from all children meeting these pre-specified screening criteria, but in practice some participants refused consent or were not offered a blood culture for other reasons.

For each participant, their age, sex, date fever started, current temperature, initial clinical diagnosis, and prior antibiotic usage were recorded at the time of fever presentation. The day of fever presentation was classified as Day 0 when counting the number of days of prior fever. Here we explore the characteristics of those who received a blood culture and those who did not to determine whether the cohort with missing data were different from those who had blood cultures performed. For those whose blood was taken for culture, we explore the factors associated with blood culture positivity.

Blood culture data were analysed using chi-square tests to determine the factors associated with blood culture collection, as well as the factors associated with positive growth of *S*. Typhi or *S*. Paratyphi. For plots of non-linear relationships, generalised additive models (GAM) were used to describe the shape of the relationship and derive confidence intervals. GAMs for binary data with logit-link functions were implemented using cubic spline penalized regression with automatic smoothness selection in R version 3.5.1, with package mcgv: Mixed GAM Computation Vehicle with Automatic Smoothness Estimation. (S1 Code & S2 Code).

For those with missing blood culture data, we used multiple imputations to create 100 imputed datasets, in which the missing outcome data were imputed based on the characteristics measured at the time of fever presentation. Each dataset contained the observed data as well as the imputed data (replacing the missing data), making 100 complete data sets in which each participant has a blood culture outcome that was either observed or imputed. For each dataset, we then summarised the number of participants with missing data that the model imputed to be blood culture positive. Less than 1% of participants had missing data for predictor variables; these individuals were excluded from the analysis.

Our multiple imputation models incorporated a MAR assumption, which can account for any systematic relationship that may be present between the characteristics of participants and the propensity for data to be missing. We tested the variables measured at the time of fever presentation to determine if there were any relationship between measured variables and the propensity for data to be missing. To conduct the multiple imputations, we used the PROC MI function in SAS version 9.4, with the logistic regression method (S3 Code). Age and days of fever were incorporated into multiple imputation models as continuous variables. Significant relationships between missingness and measured variables provide evidence to reject the MCAR assumption and proceed with MAR.

### Ethics

The study (ISRCTN43385161, https://doi.org/10.1186/ISRCTN43385161) was approved by the Oxford Tropical Research Ethics Committee (OxTREC 15–17) and the Nepal Health Research Council (Ref. no. 170/2017). Written informed consent was provided by parents/guardians of all participants.

## Results

In the first year of the study, 1903 children with eligible fevers attended surveillance clinics a total of 2392 times. Blood was drawn for culture during 1615 eligible visits (68%); 35 (2.2%) blood samples were positive for either *S*. Typhi (N = 32) or *S*. Paratyphi (N = 3). There were no children with more than one positive culture.

### Factors associated with fever presentation

The median age upon fever presentation was 4 years (IQR 2–7 years), and 53% of eligible febrile children were male. At the time of the fever visit, children had a median of 2 days of fever (IQR 1–3 days) and 52% had a current temperature of at least 38 degrees (S1 Fig and Table 1).

**Table 1:** Predictors of having blood drawn for culture and predictors of blood culture positive results in those presenting with eligible fevers of at least 2 days duration or a current temperature of 38 degrees.

Table		Number of fever presentations who had blood taken for culture	p value	Number of blood cultures positive for S.Typhi or S. Paratyphi	p value
		N (%)	(Chi-Sq)	N (%)	(Chi-Sq)
Current temperature	< 38	757/1120 (67.59%)	0.007	12/726 (1.65%)	0.200
	≥ 38	924/1272 (72.64%)		23/889 (2.59%)	
Current Temp (those with at least 2 days of fever)	<38	757/1120 (67.59%)	<0.0001	12/726 (1.65%)	0.015
	≥38	127/630 (79.84%)		19/486 (3.91%)	
Days of fever	0	105/158 (66.46%)	<0.0001	1/98 (1.02%)	0.0017
	1	316/484 (65.29%)	(trend)	3/305 (0.98%)	(trend)
	2	460/698 (65.90%)		7/441 (1.59%)	
	3	425/598 (71.07%)		11/408 (2.70%)	
	4	184/236 (77.97%)		4/177 (2.26%)	
	5	79/91 (86.81%)		2/77 (2.60%)	
	6	40/44 (90.91%)		3/39 (7.69%)	
	7	72/83 (86.75%)		4/70 (5.71%)	
Suspected typhoid	Yes	343/397 (86.40%)	<0.0001	24/333 (7.21%)	<0.0001
	No	1338/1995 (67.07%)		11/1282 (0.86%)	
Suspected upper respiratory tract infection (URTI)	Yes	775/1197 (64.75%)	<0.0001	10/746 (1.34%)	0.035
	No	906/1195 (75.82%)		25/869 (2.88%)	
Suspected lower respiratory tract infection (LRTI)	Yes	119/158 (75.32%)	0.151	0/114 (0.00%)	0.099
	No	1562/2234 (69.92%)		35/1501 (2.33%)	
Suspected urinary tract infection (UTI)	Yes	44/50 (88.00%)	0.006	2/42 (4.76%)	0.242
	No	1637/2342 (69.90%)		33/1573 (2.10%)	
Suspected diagnosis other	Yes	376/572 (65.73%)	0.006	2/364 (0.55%)	0.016
	No	1305/1820 (71.70%)		33/1251 (2.64%)	
Antibiotics Taken	No	1352/1975 (68.46%)	0.006	28/1291 (2.17%)	0.98
	Unknown	52/72 (72.22%)		1/52 (1.92%)	
	Yes	219/282 (77.66%)		5/214 (2.34%)	
Age (per year)	0	4/8 (50.00%)	<0.0001	0/4 (0.00%)	0.0009
	1	142/231 (61.47%)	(trend)	1/140 (0.71%)	(trend)
	2	255/414 (61.59%)		1/244 (0.41%)	
	3	241/344 (70.06%)		2/231 (0.87%)	
	4	195/284 (68.66%)		3/186 (1.61%)	
	5	155/226 (68.58%)		3/147 (2.04%)	
	6	141/188 (75.00%)		5/133 (3.76%)	
	7	99/133 (74.44%)		5/93 (5.38%)	
	8	106/136 (77.94%)		5/103 (4.85%)	
	9	91/105 (86.67%)		4/90 (4.44%)	
	10	60/91 (65.93%)		1/57 (1.75%)	
	11	58/75 (77.33%)		1/56 (1.79%)	
	12	42/47 (89.36%)		2/42 (4.76%)	
	13	24/31 (77.42%)		1/23 (4.35%)	
	14	34/38 (89.47%)		0/34 (0.00%)	
	15	23/27 (85.19%)		1/22 (4.55%)	
	16	10/13 (76.92%)		0/9 (0%)	
Sex	Male	935/1304 (71.70%)	0.092	21/896 (2.34%)	0.589
	Female	745/1087 (68.54%)		14/718 (1.95%)	

Note: Discrepancies between numerators in column 3 and denominators in column 5 are due to errors in sample handling or data entry.

### Factors associated with blood collection for culture upon fever presentation

**Fig 1:**
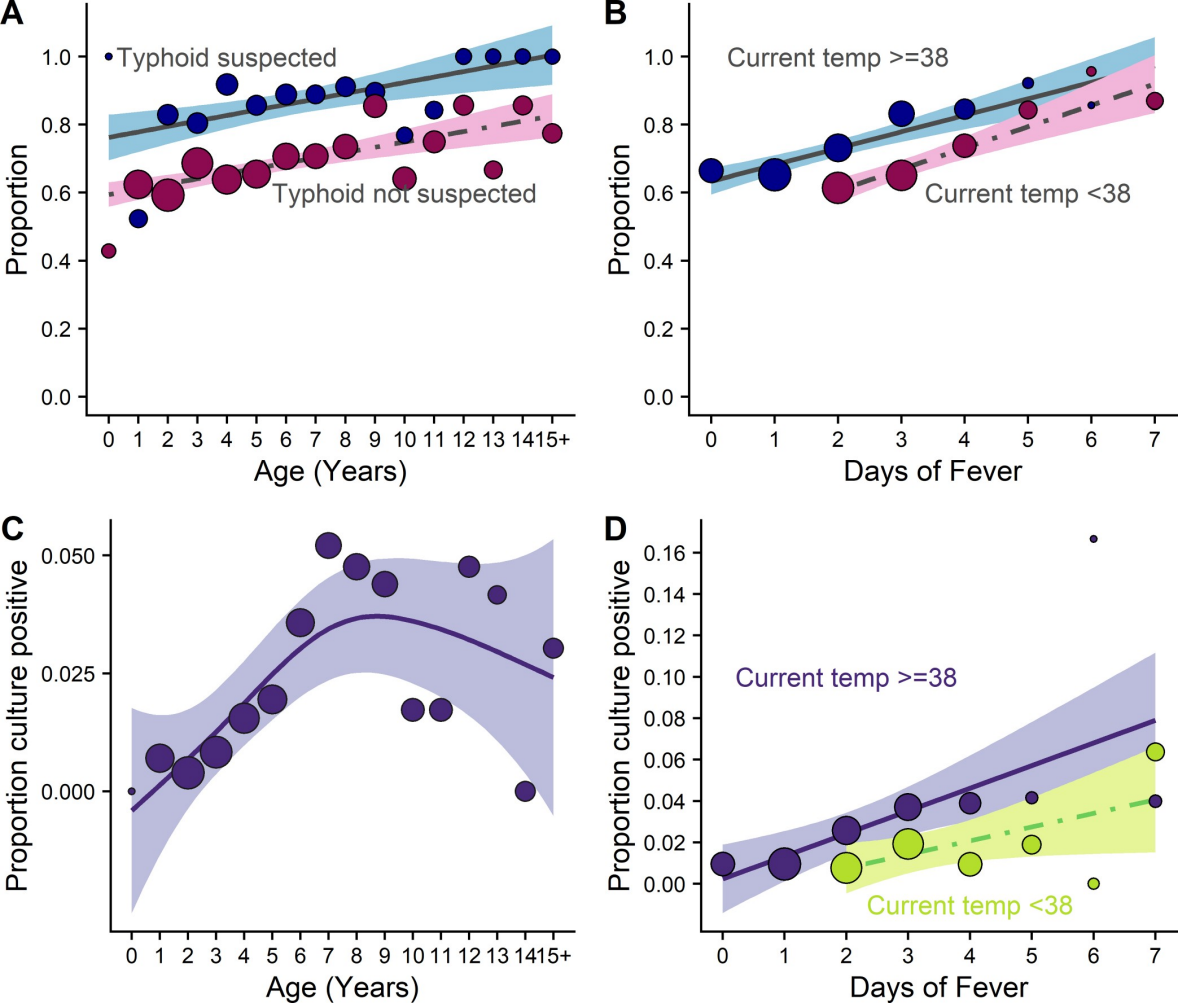
**Proportion of trial participants who have blood drawn for culture (A and B) or are blood culture positive (C and D) when presenting to fever clinics with either 2 days of self-reported fever or a current temperature of 38 degrees.** Proportion of children presenting to fever clinics who have blood drawn for culture by (A) age and clinical suspicion of typhoid (both p<0.0001), (B) fever duration (p<0.0001) and current temperature (p = 0.007). Proportion of children who have blood culture-positive enteric fever by (C) age and clinical suspicion of typhoid, (D) days with fever and current temperature. Circles represent the proportion with blood taken for each 1-year age band (A and C), or in groups according to number of days of fever (B and D). The size of the circle is proportional to the number of fever presentations in that group.

Blood was collected for culture significantly more often when there was a clinical suspicion of typhoid fever, a longer duration of fever, or when a child was older (all p< 0.0001). Children aged less than 5 years had blood collected at 65% of fever presentations, compared with 78% for children 10 years or older. Children with at least 3 days of fever had blood drawn for culture 76% of the time, while with those with fewer than 3 days of fever had blood drawn only 66% of the time (Fig 1, Table 1).

A clinical suspicion that the child had an upper respiratory tract infection (URTI) or another alternative diagnosis significantly reduced the probability of blood being collected for culture (p<0.0001 and p = 0.006, respectively). In contrast, blood was more likely to be collected when a urinary tract infection (UTI) was the initial diagnosis (p = 0.006) (Fig 1, Table 1). Clinical suspicion of a lower respiratory tract infection (LRTI) did not affect culture rates (p = 0.151).

Prior antibiotic use was associated with higher blood culture rates, whereas the sex of the child was not a significant factor (Table 1). Prior antibiotic use was also associated with duration of fever; children with at least 3 days of fever were 2.5 times more likely to report having previously taken antibiotics when presenting to clinics than those with fevers of shorter durations (RR of prior antibiotic use, ≥3 days vs < 3 days: 2.5, 95%CI; 1.99, 3.16; p<0.0001) (S2 Fig).

### Factors associated with positive blood culture

Thirty-five cases of blood culture-positive *S*. Typhi or *S*. Paratyphi fever were included in the analysis of factors associated with culture positivity. These analyses therefore had less statistical power than analyses of blood culture collection.

The predictors of positive blood cultures were similar to the predictors of blood culture collection. The strongest predictor of a positive blood culture was clinical suspicion of typhoid fever at the time of fever presentation; 24 (7.2%) blood cultures from children with clinically suspected typhoid were positive for growth of *S*. Typhi or *S*. Paratyphi, compared with only 11 (0.9%) from those who were thought not to have typhoid (p<0.0001). Nevertheless, a third of blood culture-positive fever cases were children with alternative diagnoses or for whom typhoid was not suspected as their cause of illness, even though participants were enrolled in a typhoid vaccine trial.

Of the 11 blood culture-positive cases that were not suspected of being typhoid, six were thought to have an upper respiratory tract infection (URTI), one was diagnosed as acute tonsillitis, one with a non-specific viral illness, and one was hospitalised after seven days of fever with acute gastroenteritis and dehydration. The remaining two cases had no clinical opinion recorded at the fever presentation visit. The three cases of blood culture-positive paratyphoid fever were all suspected to be URTIs.

Age was significantly associated with a positive blood culture. The proportion of positive cultures increased with age until a peak at approximately 7 years of age, after which the proportion positive plateaued (Fig 1). The lower rate of positive cultures in the younger infants likely reflects the many other causes of fevers in young children and infants.

The large majority (80%) of blood culture-positive cases were children who had not received antibiotics prior to their visit. Nevertheless, fever duration was associated with a higher proportion of positive blood cultures. Cultures were positive in 3.1% of children with at least 3 days of fever, compared with 1.3% of those with less than 3 days of fever. In those with at least 2 days of fever, 3.9% of those with a current temperature of 38 degrees or more were culture-positive compared with 1.65% of those with current temperature <38 degrees (p = 0.015) (Fig 1 and Table 1).

### Imputation of missing data

Missing data for children who did not have blood drawn for culture were imputed using a multiple imputation model. The significant associations between patient characteristics and the likelihood of blood culture data being missing provided evidence against the MCAR assumption, allowing for the MAR assumption that forms the basis of the multiple imputation model to be used.

The variables included in the imputation model were those known to be associated with blood culture sensitivity and those shown in our analysis to be associated with blood culture positivity. Specifically, the model accounted for: duration of fever, prior antibiotic use, a current fever, age, clinical suspicion of either typhoid, URTI, or another alterative diagnosis. In addition, receipt of TCV protects against typhoid fever and was therefore included[13].

**Fig 2:**
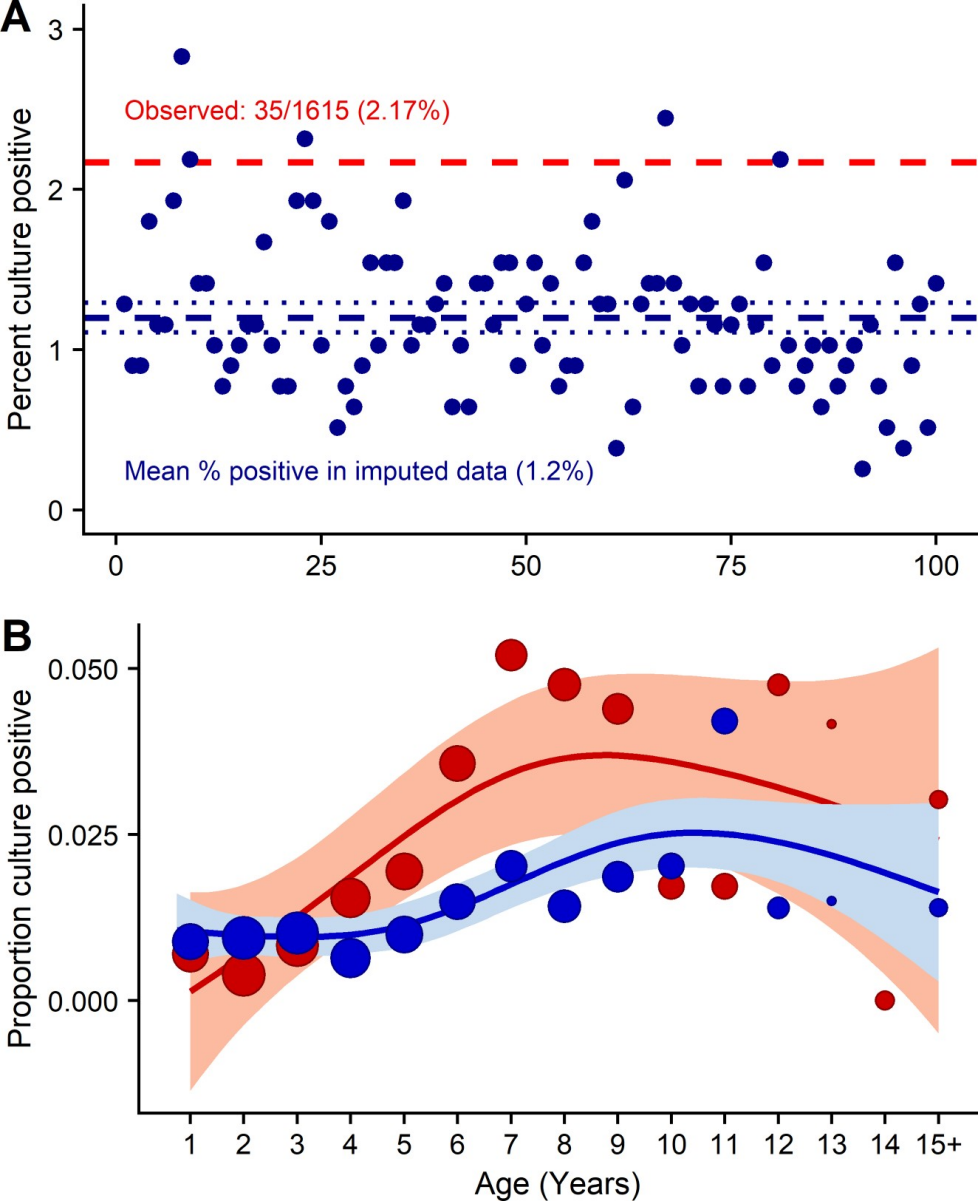
Results of 100 multiple imputation models imputing blood culture results for eligible participants presenting to fever clinics who did not have a blood culture taken. (A) Each dot shows the percentage of patients with culture-positive results in one imputed dataset, i.e. those with no blood drawn for culture (blue). The dashed blue line shows the median percent culture-positive in the imputed data, whereas the dashed red lines shows the observed percent culture-positive in those who had a blood culture taken. (B) The proportion culture-positive (observed data: red, imputed data: blue) is plotted along with the smooth spline showing that the observed data underestimate culture-positive rates in very young children less than 4 years of age and overestimate culture-positive rates in older children. Generalised additive models (GAMs) were used derive smoothed line of best fit and confidence intervals. GAMs for binary data with logit-link functions were implemented using cubic spline penalized regression with automatic smoothness selection.

When missing data were imputed, the 100 imputed trial datasets (each of size N = 2392) had a median of 44 blood culture-positive cases (IQR: 42–46), giving an overall percentage of 1.84% culture-positive. In the observed trial data, 35/1615 (2.17%) blood cultures were positive for *S*. Typhi or *S*. Paratyphi, and in the 777 participants with no blood taken, a median of 9/777 (1.16%) blood culture-positive cases were imputed. These results demonstrate the lower risk of typhoid in those who present to fever clinics but do not have blood taken for culture. The relative risk of blood culture positivity for *S*. Typhi or *S*. Paratyphi was 1.87 times higher (95% CI, 0.9–3.9) in those whose blood was collected for culture compared to those who did not have blood drawn, based on the imputed data (Fig 2).

The difference between observed and imputed data was not consistent across age groups. Fig 2B shows the proportion of cultures that were blood culture positive in the observed data (red) and the imputed data (blue). Among very young children, those with missing blood cultures were slightly more likely to have typhoid or paratyphoid fever, whereas among those aged 5–9, those who had missing blood cultures were less likely to have typhoid. The ratios comparing observed and imputed data by age group are shown in Table 2.

**Table 2:** Comparison of observed and imputed data from 100 multiple imputation models, by age group.

Age group	Observed cases (out of 1615)	% culture positive in observed data	Number of imputed culture positive cases (out of 777) (95% CI)	% culture positive in imputed (missing) data (95% CI)	Inflation due to missing data (% positive observed/ % positive imputed)
**< 2y**	1	0.69	0.86 (0.65, 1.07)	0.91 (0.68, 1.13)	0.77 (0.61, 1.01)
**2-4y**	6	0.91	3.39 (2.96, 3.82)	0.89 (0.76, 1.00)	1.02 (0.91, 1.20)
**5-9y**	22	3.89	3.17 (2.81, 3.53)	1.43 (1.28, 1.59)	2.72 (2.45, 3.04)
**10–15**	6	2.47	1.91 (1.62, 2.20)	2.42 (2.04, 2.79)	1.02 (0.89, 1.21)
**Total**	**35**		**9**		

## Discussion

In our passive surveillance programme for typhoid fever in Nepal, children presenting with eligible fevers but not receiving a blood culture had a lower risk of typhoid fever. These patients were younger, had fever for a shorter duration of time, and were more likely to have an alternative initial diagnosis such as an upper respiratory tract infection or viral illness. Although these patients were less likely to be blood culture positive, there is little doubt that positive cases were missed because this group did not have blood taken for diagnosis; thus, their exclusion from incidence estimates would lead to under-estimation of the true burden of disease. In contrast, if we assumed that those with missing data were just as likely to be blood culture-positive when adjusting incidence estimates, we would over-estimate the disease burden, particularly among those aged 5–9 years. Thus, competing sources of bias need to be accounted for appropriately in analyses.

The accuracy of clinical opinion in diagnosing typhoid fever in this high-incidence setting means that those who are suspected of having typhoid are more likely to have blood taken for culture and also more likely to have a positive blood culture. Nevertheless, a substantial proportion of culture-positive cases occurred among those with an alternative clinical diagnosis, with URTI being the most common alternative diagnosis despite the fact that overall individuals with clinical suspicion of URTI were less likely to have blood drawn for culturing. Parental opinion may also play a part in whether or not blood is taken for diagnosis, with parents of younger children being more reticent to have blood drawn for culture in Nepal. In our study, we estimated that those who had blood cultures performed were 1.87 times more likely to be positive for *Salmonella* than those without blood cultures.

For double-blind individually randomised clinical trials, missed cases should not introduce bias into vaccine efficacy estimates, since the vaccine is randomly allocated to individuals in the population, and the decision to collect blood for culturing does not depend on vaccination status when both researchers and patients are unaware as to whether they received TCV or the control vaccine. However, missed cases will decrease the power to detect a significant vaccine effect.

For surveillance studies aiming to estimate incidence, adjusting for missing data can result in under- or over-estimates of incidence. The “WHO surveillance standard for typhoid and other invasive salmonellosis”[14] defines a suspected typhoid case as someone with at least 3 days of fever (out of 7 consecutive days), and recommends blood cultures be taken for this group. Surveillance of typhoid fever is resource intensive, and very few blood cultures taken are positive for *S*. Typhi. The 3-day criterion increases the probability that a blood culture will be positive and focusses surveillance effort on these potentially more severe cases. Our data show that positive blood cultures are more than twice as likely to be obtained from patients with at least 3 days of fever compared to those with less than 3 days, a finding which supports the WHO recommendation. However, a third of our blood culture-positive cases were in those with less than 3 days of fever. These patients would be missed in surveillance programmes using the 3-day criterion, resulting in underestimation of the true incidence of disease. In addition, antibiotic use is more than twice as likely in children with at least 3 days of fever in our data, which may compromise the sensitivity of blood culture. Bacterial growth is less likely to occur in blood samples taken after antibiotic treatment has commenced. The large majority of our culture-positive cases did not report prior antibiotic use. As self-treatment with antibiotics from local pharmacies continues to increase in many low- and middle-income countries, the usefulness of the 3-day criterion for blood taking may diminish. Our results show that those with 2 days of fever combined with a current temperature of at least 38 degrees are just as likely to be blood culture positive as those with 3 or more days of fever. This group has a lower rate of antibiotic use; therefore, extension of surveillance programmes to capture these patients may be beneficial.

The data for this study came from an ongoing typhoid conjugate vaccine trial in children. Our findings may not be relevant to blood culture collection and disease incidence in adults. In addition, our data reflect attitudes around diagnosis and treatment in the Lalitpur Metropolitan Region of Nepal, and therefore our results may not be generalizable to other settings. Attitudes, disease incidence, and access to health facilities vary from country to country. Similar data and analyses from other countries are needed in order to understand inter-country variation.

Not all febrile patients who present to a typhoid fever surveillance clinic will receive a blood culture, even when eligible. Clinical opinion on the cause of the fever may play a large part in the decision to encourage blood draws for culture, resulting in a group of patients with missing blood culture data who are less likely to be blood culture positive. Crude typhoid incidence estimates should be adjusted for both the proportion of cases that go undetected due to missing blood cultures as well as the lower likelihood of culture-positivity in the group with missing data.

## Supporting information

S1 Checklist STROBE checklist. (DOC)

S1 Fig Age and number of days with fever at the time of study enrolment or fever presentation. A: Age of children at time of enrolment into the trial, B: Age of children at the time of fever presentation, C: Number of days with fever prior to the day of fever presentation (fevers which started on the same day as the fever presentation were classed as 0 days of fever), D: Number of days with fever in those with a current temperature of 38 degrees or more at the time of fever presentation.
(PNG)

S2 Fig Longer duration of fever is associated with greater prior antibiotic use in children presenting to fever clinics. Size of circles is proportional to the number of fevers. Smooth line is from a generalised additive model with cubic spline smooth; the shaded region represents the 95% confidence interval.
(PNG)

S1 Code Missing data fever presentations PLosNTD upload.R. R code for the analysis of fever presentation data (Fig 1A and 1B)
(R) Click here for additional data file.

S2 Code Missing blood culture data PLoSNTD upload.R. R code for the analysis of blood culture data (Fig 1C & 1D and Fig 2B)
(R) Click here for additional data file.

S3 Code Multiple imputation PLoS NTD upload.sas. SAS code for performing multiple imputations.
(SAS)

S1 Data Out_mi.csv. Data set of the results of the multiple imputations.
(CSV)

S2 Data TyVACNepalPLoSNTD.csv. Dataset of fever presentation and blood culture data.
(CSV)
