# Supplementary figures and images for: Under-detection of blood culture-positive enteric fever cases: The impact of missing data and methods for adjusting incidence estimates
Source: PLoS Negl Trop Dis. 2020 Jan 16;14(1):e0007805. doi: 10.1371/journal.pntd.0007805 (PMC6964825; doi:10.1371/journal.pntd.0007805)

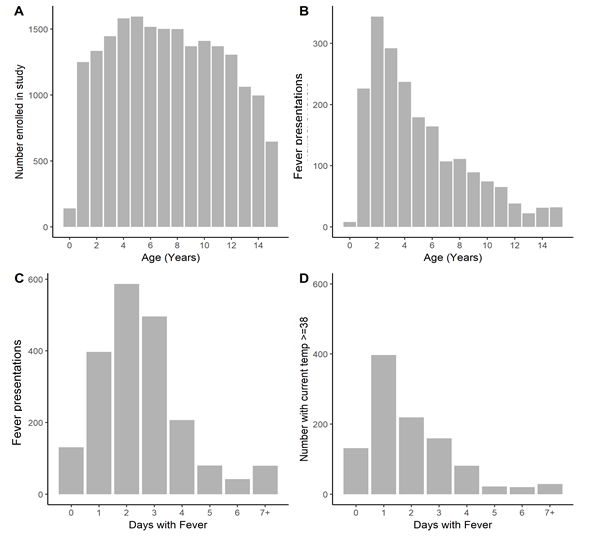

Supplement: S1 Fig — A: Age of children at time of enrolment into the trial, B: Age of children at the time of fever presentation, C: Number of days with fever prior to the day of fever presentation (fevers which started on the same day as the fever presentation were classed as 0 days of fever), D: Number of days with fever in those with a current temperature of 38 degrees or more at the time of fever presentation. (PNG) [file pntd.0007805.s002.png]

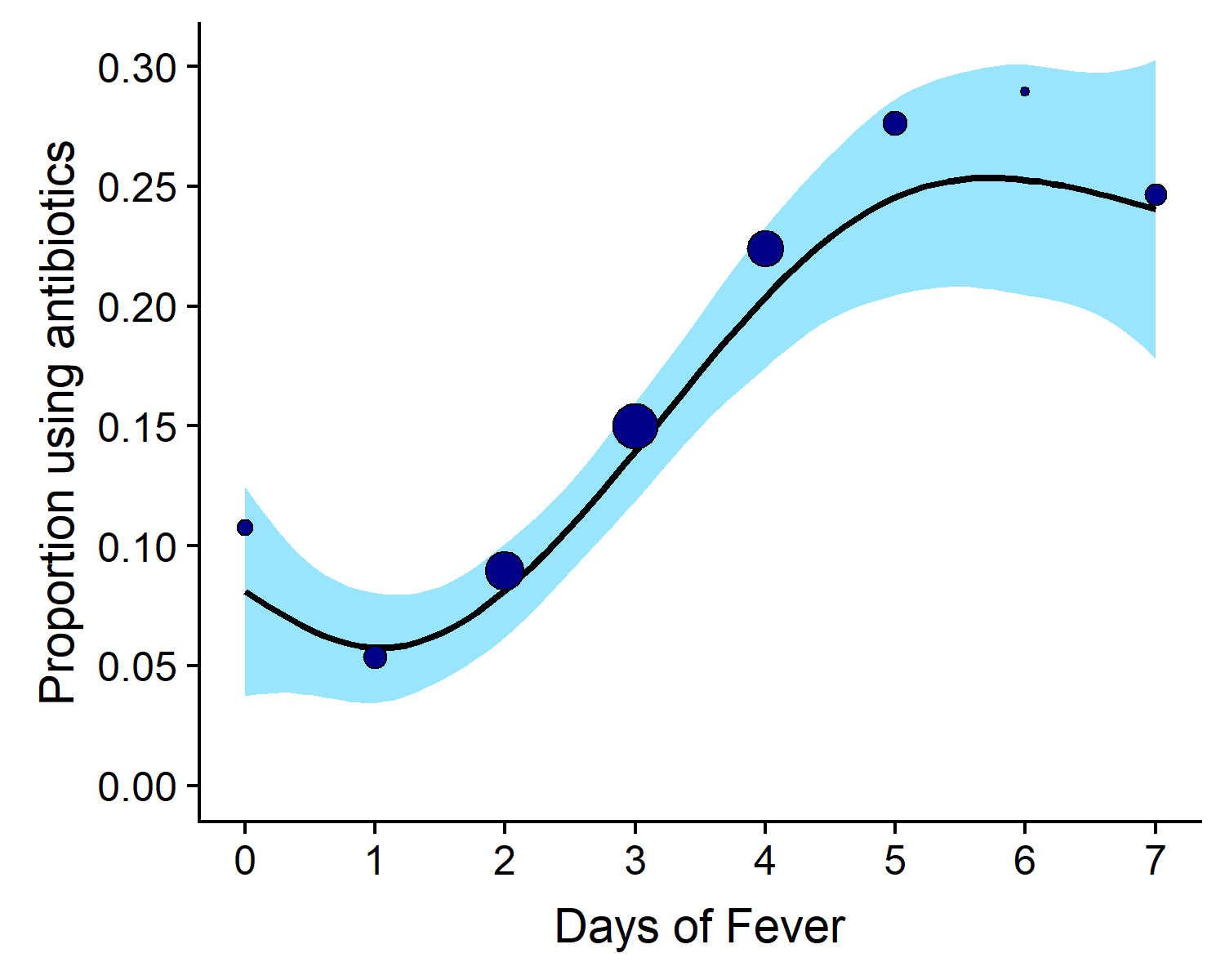

Supplement: S2 Fig — Size of circles is proportional to the number of fevers. Smooth line is from a generalised additive model with cubic spline smooth; the shaded region represents the 95% confidence interval. (PNG) [file pntd.0007805.s003.png]
